# Routes of Marijuana Use — Behavioral Risk Factor Surveillance System, 22 U.S. States and Two Territories, 2022

**DOI:** 10.15585/mmwr.mm7412a1

**Published:** 2025-04-10

**Authors:** Zerleen S. Quader, Douglas R. Roehler, Alana M. Vivolo-Kantor, Jean Y. Ko

**Affiliations:** ^1^Division of Overdose Prevention, National Center for Injury Prevention and Control, CDC; ^2^Epidemic Intelligence Service, CDC.

**Summary**

**What is already known about this topic?**

Cannabis policies, availability, products, and use patterns in the United States have changed during the last several years. Historically, smoking has been the most common route of cannabis use; however, other routes of use are increasing.

**What is added by this report?**

In 2022, 15.3% of adults reported current cannabis use, approximately 80% of whom reported smoking. Eating, vaping, and dabbing (inhaling heated concentrated cannabis) were also common, and approximately one half of respondents reported multiple routes of use. Vaping and dabbing were most prevalent among adults aged 18–24 years.

**What are the implications for public health practice?**

Continued surveillance of routes of cannabis use and use patterns might be helpful to understanding health outcomes in the evolving cannabis marketplace.

## Abstract

Access to and use of cannabis in the United States has increased as new product types emerge in the marketplace, and as additional states legalize its use for medical and nonmedical purposes. To tailor education messages for preventing adverse health effects of cannabis use, understanding the routes of use of these products in the general population is important. The 2022 Behavioral Risk Factor Surveillance System included a newly revised optional marijuana module comprising questions on marijuana routes of use among adults aged ≥18 years who used marijuana during the past 30 days (current use). Twenty-two states and two territories administered the optional marijuana module in 2022. Weighted prevalences (with 95% CIs) of current and daily or near-daily marijuana use, as well as prevalence of each route of use, were reported overall and by demographic characteristics and, among women aged ≤49 years, by pregnancy status. Among the 15.3% of respondents who reported current marijuana use, smoking was the most frequent route (79.4%), followed by eating (41.6%), vaping (30.3%), and dabbing (inhaling heated concentrated cannabis) (14.6%). Vaping and dabbing were most prevalent among persons aged 18–24 years. Intervention measures intended for persons who smoke cannabis are important; however, understanding health outcomes associated with other routes of use might have substantial public benefit.

## Introduction

At the federal level, cannabis remains classified as a Schedule I substance under the Controlled Substances Act, making distribution of cannabis a federal offense. However, as of April 2025, 39 states, three territories, and the District of Columbia (DC) have legalized cannabis* use for state-defined qualifying medical conditions, and 24 states, two territories, and DC have legalized nonmedical adult cannabis use (*1*). In recent decades, the perception of risk associated with cannabis use has decreased; cannabis products containing higher concentrations of tetrahydrocannabinol (THC), the psychoactive compound found in cannabis, have become more intoxicating, and routes of use (more commonly known as routes of administration) have evolved (*2*).

As the availability and types of cannabis products expand, less is known about how persons consume cannabis. Historically, cannabis has most often been smoked; however, additional routes of use are available, including oral ingestion, vaping, and more recently, dabbing (i.e., inhalation of highly concentrated THC-based oils often heated using a blowtorch) (*3*,*4*). Different routes of use might increase the risk for certain health effects; examples include an increased risk for lung injury associated with potential contaminants when vaping cannabis,^†^ acute psychosis from dabbing highly concentrated THC products, or overconsumption of cannabis when ingesting edibles (*4*).

Surveillance is important to better understand routes of cannabis use and frequency of use, especially given the rapid shifts in the cannabis marketplace. Limited information is available on the current prevalence of the most common routes of cannabis use. U.S. studies on routes of cannabis use might be outdated or include small sample sizes and are frequently single-state samples (*3*,*5*). This study analyzed data from the optional marijuana module administered in 22 U.S. states and two territories as part of the 2022 Behavioral Risk Factor Surveillance System (BRFSS) to measure the prevalence of routes of cannabis use among adults aged ≥18 years.

## Methods

### Data Source and Primary Measures

BRFSS is an annual, state-based landline and cellular telephone survey of health behaviors and conditions of noninstitutionalized adults aged ≥18 years in all 50 U.S. states, DC, and three territories.^§^ The median combined response rate for BRFSS in 2022 was 45.1% for all states and territories, ranging from 22% to 58% in the jurisdictions included in this study^¶^ (*6*).

In 2022, a total of 22 U.S. states and two territories** included a revised (2022) optional marijuana module that asked about current marijuana use and routes of use.^††^ This was the first optional marijuana module administered since 2016 that permitted selection of multiple routes of use. In the revised 2022 module, respondents who reported past 30-day use were also asked to indicate all routes of marijuana use and primary route of use during the previous 30 days (during 2017–2021, the marijuana modules only permitted selection of one primary route of use).

Current marijuana use was defined as any reported use during the past 30 days, and daily or near-daily use (daily use) was defined as reported use ≥20 times during the past 30 days. Current and daily marijuana use and routes of use were measured by age group (18–24, 25–34, 35–44, 45–54, 55–64, and ≥65 years), sex (female or male), race and ethnicity (non-Hispanic American Indian or Alaska Native [AI/AN], non-Hispanic Asian, non-Hispanic Black or African American, non-Hispanic Native Hawaiian or Pacific Islander [NH/PI], non-Hispanic White; Hispanic or Latino [Hispanic], and non-Hispanic multiracial persons), highest level of education attained (less than high school, high school diploma or general educational development certificate, some college, or college degree), and pregnancy status^§§^ (pregnant or not pregnant).

### Statistical Analysis

Prevalence and 95% CIs of current and daily marijuana use and routes of use were reported overall and by age group, sex, race and ethnicity, education level, and pregnancy status. Rao-Scott chi-square tests were used to identify differences across sociodemographic characteristics, with p-values <0.05 considered statistically significant. Sample weights and design variables were used to account for the complex survey design.^¶¶^ Analyses were conducted using R (version 4.3.2, R Foundation). This activity was reviewed by CDC, deemed not research, and was conducted consistent with applicable federal law and CDC policy.***

## Results

The study population comprised 138,625 respondents, including 14,044 (15.3%) who reported current marijuana use, and 6,848 (7.9%) who reported daily use (Table 1). Both current and daily marijuana use were most prevalent among adults aged 18–24 years (25.9% and 13.4%, respectively), males (18.0% and 9.5%, respectively), non-Hispanic multiracial adults (24.7% and 14.0%, respectively), AI/AN adults (20.7% and 14.0%, respectively), those with a high school diploma or less (17.2%–17.4% and 10.1%–12.1%, respectively), and women who were not pregnant (19.5% and 10.1%, respectively).

**TABLE 1:** Prevalence of current* and daily or near-daily^†^ marijuana^§^ use, overall and across sociodemographic characteristics — Behavioral Risk Factor Surveillance System, 22 U.S. states and two territories,**^¶^** 2022

Characteristic	Total no.	Current marijuana use*			Daily or near-daily marijuana use^†^		
		No.	Weighted % (95% CI)	p-value**	No.	Weighted % (95% CI)	p-value**
**Overall**	**138,625**	**14,044**	**15.3 (14.9–15.7)**	**—**	**6,848**	**7.9 (7.6–8.2)**	**—**
**Age group, yrs**							
18–24	**8,063**	1,626	25.9 (24.1–27.9)	<0.001	779	13.4 (11.9–15.0)	<0.001
25–34	**13,968**	2,579	24.2 (22.7–25.6)		1,345	13.0 (11.9–14.1)	
35–44	**17,881**	2,635	19.2 (18.1–20.4)		1,370	10.6 (9.7–11.6)	
45–54	**20,393**	2,089	12.9 (12.0–13.9)		1,035	6.6 (6.0–7.4)	
55–64	**26,107**	2,490	11.3 (10.5–12.2)		1,145	5.5 (5.0–6.2)	
≥65	**52,213**	2,625	6.2 (5.7–6.7)		1,174	2.6 (2.3–2.9)	
**Sex**							
Female	**73,892**	6,047	12.8 (12.2–13.4)	<0.001	2,824	6.4 (6.0–6.9)	<0.001
Male	**64,733**	7,997	18.0 (17.3–18.7)		4,024	9.5 (8.9–10.0)	
Race and ethnicity**							
AI/AN	**2,092**	331	20.7 (17.3–24.7)	<0.001	207	14.0 (11.0–17.5)	<0.001
Asian	**5,282**	240	5.9 (4.7–7.4)		86	1.7 (1.2–2.6)	
Black or African American	**11,245**	1,117	19.0 (17.3–20.8)		597	10.4 (9.2–11.8)	
NH/PI	**1,732**	211	17.1 (11.7–24.4)		89	6.5 (4.2–10.0)	
White	**103,965**	10,427	15.0 (14.5–15.5)		4,964	7.6 (7.2–7.9)	
Hispanic or Latino	**10,414**	1,099	13.4 (12.2–14.7)		568	7.1 (6.2–8.1)	
Multiracial	**3,895**	619	24.7 (21.5–28.2)		337	14.0 (11.5–16.9)	
**Education**							
Less than HS	**7,516**	844	17.4 (15.6–19.4)	<0.001	525	12.1 (10.5–13.9)	<0.001
HS diploma or GED	**35,221**	4,001	17.2 (16.3–18.1)		2,266	10.1 (9.4–10.8)	
Some college	**38,245**	4,320	16.9 (16.0–17.7)		2,207	8.5 (7.9–9.2)	
College degree	**57,031**	4,862	11.5 (11.0–12.1)		1,840	4.1 (3.8–4.4)	
**Pregnancy status^††^**							
Pregnant	**706**	43	6.6 (4.2–10.3)	<0.001	21	4.2 (2.3–7.6)	0.003
Not pregnant	**23,254**	3,431	19.5 (18.5–20.6)		1,646	10.1 (9.3–11.0)	

**Abbreviations:** AI/AN = American Indian or Alaska Native; GED = general educational development certificate; HS = high school; NH/PI = Native Hawaiian or Pacific Islander.

* Current use is defined as any use during the past 30 days.

^†^ Daily or near-daily use is defined as use ≥20 times during the past 30 days.

^§^ The term marijuana, rather than cannabis, is used when referring to survey findings to align with the Behavioral Risk Factor Surveillance System optional marijuana module. In this report, both terms refer to the dried flowers, leaves, stems, and seeds of the cannabis plant and do not include hemp-based or cannabidiol-only products. All table comparison p-values are based on Rao-Scott chi-square tests.

^¶^ Reflects noninstitutionalized adults (aged ≥18 years) in Connecticut, Delaware, Hawaii, Illinois, Indiana, Kansas, Maine, Maryland, Massachusetts, Michigan, Mississippi, Montana, Nebraska, Nevada, New Mexico, North Dakota, Ohio, Oklahoma, Oregon, Virginia, Wisconsin, Wyoming, Guam, and the U.S. Virgin Islands.

** Persons of Hispanic or Latino (Hispanic) origin might be of any race but are categorized as Hispanic; all racial groups are non-Hispanic.

**^††^** Pregnancy status only asked of women aged ≤49 years.

Among adults who reported current cannabis use, smoking was the most prevalent route of use (79.4%), followed by eating (41.6%), vaping (30.3%), and dabbing (14.6%) (Table 2). Vaping and dabbing were most prevalent among adults aged 18–24 years (44.7% and 28.4%, respectively) and among NH/PI adults (51.7% and 42,9%, respectively). Dabbing was also more prevalent among AI/AN adults (29.3%), and adults with less than a high school diploma (23.0%). A majority of respondents reported smoking as their primary route of use (62.4%; 95% CI = 60.2%–64.6%), followed by vaping (16.8%; 95% CI = 15.1%–18.6%) and eating (14.2%; 95% CI = 12.8%–15.8%).

**TABLE 2:** Prevalence of routes of marijuana use among persons who reported current marijuana use,*^,^^†^ overall and across sociodemographic characteristics — Behavioral Risk Factor Surveillance System, 22 U.S. states and two territories,**^§^** 2022

Characteristic	Route of marijuana use									
	Smoking		Eating		Vaping		Dabbing^¶^		Other	
	Weighted % (95% CI)	p-value	Weighted % (95% CI)	p-value	Weighted % (95% CI)	p-value	Weighted % (95% CI)	p-value	Weighted % (95% CI)	p-value
**Overall**	**79.4 (78.2–80.5)**	**—**	**41.6 (40.1–43.2)**	**—**	**30.3 (28.8–31.9)**	**—**	**14.6 (13.5–15.8)**	**—**	**6.0 (5.3–6.9)**	**—**
**Age group, yrs**										
18–24	86.1 (83.2–88.5)	<0.001	36.5 (32.7–40.5)	<0.001	44.7 (40.6–48.9)	<0.001	28.4 (24.7–32.4)	<0.001	6.2 (4.3–8.5)	0.887
25–34	81.7 (78.9–84.1)		42.2 (38.9–45.6)		36.6 (33.4–40.0)		16.8 (14.6–19.3)		6.3 (4.6–8.5)	
35–44	77.0 (74.2–79.6)		48.3 (44.9–51.7)		30.9 (27.7–34.3)		13.4 (11.0–16.1)		5.2 (3.8–6.9)	
45–54	76.0 (72.7–79.0)		46.4 (42.7–50.3)		23.4 (20.4–26.6)		11.2 (9.0–13.9)		6.6 (5.1–8.3)	
55–64	79.8 (76.9–82.4)		34.6 (31.2–38.1)		16.4 (13.7–19.4)		4.6 (3.3–6.5)		6.2 (4.3–8.5)	
≥65	69.0 (65.3–72.5)		39.1 (35.4–43.0)		11.9 (9.8–14.4)		1.6 (1.0–2.5)		6.0 (4.6–7.8)	
**Sex**										
Female	74.2 (72.2–76.1)	<0.001	46.4 (43.9–48.8)	<0.001	29.3 (27.0–31.7)	0.24	10.9 (9.4–12.7)	<0.001	6.4 (5.4–7.6)	0.375
Male	83.3 (81.8–84.7)		38.0 (36.0–40.0)		31.1 (29.2–33.1)		17.4 (15.8–19.2)		5.8 (4.7–6.9)	
Race and ethnicity**										
AI/AN	87.2 (77.4–93.2)	<0.001	34.8 (26.7–44.0)	<0.001	36.8 (27.8–46.8)	<0.001	29.3 (21.0–39.2)	<0.001	8.5 (3.9–15.8)	0.033
Asian	72.9 (61.0–82.3)		31.5 (22.2–42.4)		39.0 (28.1–51.2)		15.9 (8.3–28.5)		4.6 (0.9–13.0)	
Black or African American	89.6 (86.5–92.0)		30.9 (26.3–35.9)		19.8 (15.9–24.4)		8.0 (5.6–11.2)		4.8 (2.5–8.2)	
NH/PI	78.5 (44.2–94.4)		34.6 (19.8–53.2)		51.7 (32.3–70.6)		42.9 (23.4–65.0)		5.8 (0.7–19.9)	
White	76.5 (75.0–77.9)		44.7 (43.0–46.5)		31.2 (29.4–32.9)		14.6 (13.2–16.0)		5.5 (4.8–6.3)	
Hispanic or Latino	84.5 (81.0–87.5)		39.4 (32.0–47.2)		33.2 (28.6–38.2)		18.9 (15.0–23.5)		9.0 (6.1–12.6)	
Multiracial	79.5 (72.2–85.3)		40.2 (35.3–45.3)		34.6 (27.3–42.8)		16.2 (11.2–22.8)		10.3 (6.0–16.1)	
**Education**										
Less than HS	92.1 (88.9–94.4)	<0.001	28.3 (23.3–33.9)	<0.001	28.8 (23.7–34.5)	0.055	23.0 (18.1–28.8)	<0.001	6.1 (3.9–9)	0.82
HS diploma or GED	86.0 (84.0–87.7)		33.9 (31.3–36.6)		32.2 (29.5–35.1)		20.2 (17.9–22.7)		6.6 (5.1–8.3)	
Some college	80.8 (78.6–82.9)		40.9 (38.1–43.6)		31.6 (29.0–34.4)		12.5 (10.9–14.4)		5.7 (4.5–7.2)	
College degree	62.7 (60.2–65.2)		59.0 (65.5–61.5)		26.8 (24.6–29.1)		6.5 (5.4–8.0)		5.9 (4.7–7.2)	
**Pregnancy status^††^**										
Pregnant	72.0 (46.2–88.5)	0.65	36.0 (17.6–59.6)	0.38	23.4 (9.1–48.2)	0.34	12.2 (4.5–29.0)	0.82	7.5 (1.3–33.3)	0.85
Not pregnant	76.8 (74.3–79.1)		46.5 (43.4–49.5)		34.5 (31.6–37.5)		13.6 (11.6–15.9)		6.3 (5.1–7.8)	

**Abbreviations:** AI/AN = American Indian or Alaska Native; GED = general educational development certificate; HS = high school; NH/PI = Native Hawaiian or Pacific Islander.

* The term marijuana, rather than cannabis, is used when referring to survey findings to align with the Behavioral Risk Factor Surveillance System optional marijuana module. In this report, both terms refer to the dried flowers, leaves, stems, and seeds of the cannabis plant and do not include hemp-based or cannabidiol-only products. All table comparison p-values are based on Rao-Scott chi-square tests.

**^†^** Current use is defined as any use during the past 30 days.

^§^ Reflects noninstitutionalized adults (aged ≥18 years) in Connecticut, Delaware, Hawaii, Illinois, Indiana, Kansas, Maine, Maryland, Massachusetts, Michigan, Mississippi, Montana, Nebraska, Nevada, New Mexico, North Dakota, Ohio, Oklahoma, Oregon, Virginia, Wisconsin, Wyoming, Guam, and the U.S. Virgin Islands.

^¶^ Inhalation of highly concentrated tetrahydrocannabinol-based oils, often heated using a blowtorch.

** Persons of Hispanic or Latino (Hispanic) origin might be of any race but are categorized as Hispanic; all racial groups are non-Hispanic.

**^††^** Pregnancy status only asked of women aged ≤49 years.

Approximately one half of adults who currently use cannabis reported two or more routes of use (46.7%; 95% CI = 45.1%–48.3%). Among adults who reported current cannabis use and two or more routes of use, the most prevalent combinations were smoking and eating (55.2%; 95% CI = 52.7%–57.6%) and smoking and vaping (54.5%; 95% CI = 52.1%–56.9%) (Figure).

**FIGURE:**
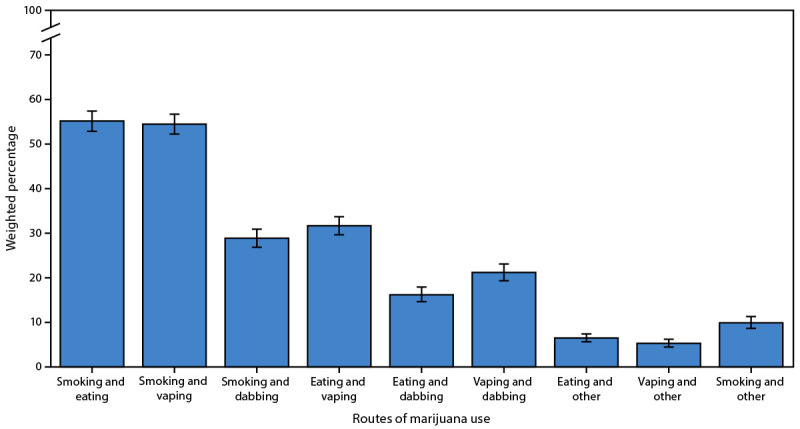
Combinations of routes of marijuana use among respondents who reported current use and two or more routes of use (N = 5,813) — Behavioral Risk Factor Surveillance System, 22 U.S. states and two territories,*^,^^†^^,^^§^ 2022 * 95% CIs indicated by bars. ^†^ The term marijuana, rather than cannabis, is used when referring to survey findings to align with the Behavioral Risk Factor Surveillance System optional marijuana module. In this report, both terms refer to the dried flowers, leaves, stems, and seeds of the cannabis plant and do not include hemp-based or cannabidiol-only products. ^§^ Noninstitutionalized adults (aged ≥18 years) in Connecticut, Delaware, Hawaii, Illinois, Indiana, Kansas, Maine, Maryland, Massachusetts, Michigan, Mississippi, Montana, Nebraska, Nevada, New Mexico, North Dakota, Ohio, Oklahoma, Oregon, Virginia, Wisconsin, Wyoming, Guam, and the U.S. Virgin Islands.

## Discussion

In this analysis of the BRFSS optional marijuana module, 15.3% of adults reported current marijuana use. Among adults aged 18–24 and 25–34 years, approximately one in four reported current marijuana use, and approximately one in eight reported daily use. Current marijuana use was lower among pregnant women than among those who were not pregnant, similar to findings from other national studies (*7*). Approximately four in five adults with current cannabis use reported smoking; other routes of use, including eating, vaping, and dabbing, were also common. Approximately one half of adults with current cannabis use reported multiple routes of use. 

Compared with 2016 BRFSS data in 12 states, the prevalences of eating and vaping marijuana were each higher in 2022, as was the prevalence of reporting multiple routes of use (*3*). These differences might reflect a shift in use patterns and might also reflect the larger sample and inclusion of different states in this analysis. Monitoring these changes is important because each route of use is associated with unique health risks. For example, the wider availability of edibles has been associated with increased accidental pediatric ingestion (*2*).

Vaping and dabbing were most common among young adults aged 18–24 years. Trends in both of these routes of use have increased among adolescents and young adults (*2*,*8*). This shift in routes of use among younger persons could lead to exposure to higher concentrations of THC at an age when brain development is still occurring, and thus increase the risk for cannabis use disorder, injuries, or acute psychosis (*2*). In addition, those who consume cannabis through vaping can also be exposed to potentially harmful contaminants or adulterants, as was the case during the 2019 e-cigarette, or vaping, product use–associated lung injury outbreak that was strongly linked with vitamin E acetate, an additive in some THC-containing vapes (*9*). Dabbing also often requires the use of a blowtorch, which might increase the risk for burn injuries (*4*). AI/AN and NH/PI adults also reported the highest prevalences of dabbing (42.9% and 29.3%, respectively). Therefore, increased efforts to decrease dabbing among these two populations might decrease the risk for the associated potential adverse health effects.

### Limitations

The findings in this report are subject to at least three limitations. First, the BRFSS optional marijuana module was not administered in all jurisdictions; therefore, this study sample is not representative of the entire U.S. adult population. Second, data are self-reported, which might lead to underestimation of prevalence estimates if respondents were influenced by social desirability. Finally, questions about routes of use had not been consistently asked across previous BRFSS survey years or asked consistently across the same jurisdictions every survey year. Therefore, it is not possible to examine trends in routes of use, and comparisons of results to those obtained in previous years might reflect changes in sampling rather than only changes in prevalence.

### Implications for Public Health Practice

Given the prevalence of cannabis smoking, eating, vaping, and dabbing, public health–related messaging specific to these routes of use can help guide persons about potential risks. Messaging can focus on the risks related to each of these routes of use, such as exposure to contaminants or adulterants with vaping, or exposure to high concentrations of THC from ingestion, vaping, and dabbing. These findings can be used to guide tailored educational messaging for cannabis-related harms. Continued surveillance of the frequency of cannabis use, routes of use, and concentrations of THC present in different products consumed is needed to understand health outcomes in the changing cannabis marketplace and protect those who use cannabis in its various forms.

## Footnotes

* Cannabis (e.g., marijuana, weed, pot, or bud) refers to the dried flowers, leaves, stems, and seeds of the cannabis plant and does not include hemp-based or cannabidiol-only products. Because the term marijuana is used in the Behavioral Risk Factor Surveillance System optional marijuana module, it is used in this report when referring to the survey results.

†
https://archive.cdc.gov/#/details?url=https://www.cdc.gov/tobacco/basic_information/e-cigarettes/severe-lung-disease.html

§
https://www.cdc.gov/brfss/

¶ https://www.cdc.gov/brfss/annual_data/2022/pdf/Overview_2022-508.pdf

** Connecticut, Delaware, Hawaii, Illinois, Indiana, Kansas, Maine, Maryland, Massachusetts, Michigan, Mississippi, Montana, Nebraska, Nevada, New Mexico, North Dakota, Ohio, Oklahoma, Oregon, Virginia, Wisconsin, Wyoming, Guam, and the U.S. Virgin Islands.

††
https://www.cdc.gov/brfss/questionnaires/modules/category2022.htm

§§ Only asked of women aged ≤49 years.

¶¶
https://www.cdc.gov/brfss/annual_data/2022/pdf/Complex-Sampling-Weights-and-Preparing-Module-Data-for-Analysis-2022-508.pdf

*** 45 C.F.R. part 46, 21 C.F.R. part 56; 42 U.S.C. Sect. 241(d); 5 U.S.C. Sect. 552a; 44 U.S.C. Sect. 3501 et seq.
