# *Erratum*: Vol. 74, No. 12

**DOI:** 10.15585/mmwr.mm7414a5

**Published:** 2025-04-24

The report, “Routes of Marijuana Use — Behavioral Risk Factor Surveillance System, 22 U.S. States and Two Territories, 2022” contained several errors. 

On page 198, the first two sentences of the Introduction should have read, “**At the federal level, cannabis remains classified as a Schedule I substance under the Controlled Substances Act, making distribution of cannabis a federal offense. However, as of April 2025, 39** states, three territories, and the District of Columbia (DC) have legalized cannabis* use for state-defined qualifying medical conditions, and 24 states, two territories, and DC have legalized nonmedical adult cannabis use (*1*).”

On page 201, in Table 1, under the column heading “Daily or near-daily marijuana use,” under the subheading “No.,” the values for “Age group, yrs” should have read, “**779**; **1,345**; **1,370**; **1,035**; **1,145**; and **1,174**.”

